# Social Problems of the Day

**Published:** 1907-09-21

**Authors:** 


### SOCIAL PROBLEMS OF THE DAY.

### WILL UTOPIA EXCLUDE CHARITY?

II. Charity as Taster to Humanity.

In a previous article the ethics of Charity were examined in relation to countries in which the large majority of workers are able to earn no more than just sufficient for their own needs.

But let it be granted that the community has reached such a level of prosperity that the wage earners are in a position to meet all claims arising upon them for distress in their midst. How will Charity stand then? Will it be an offence to stretch out a hand to rescue a neighbour from misfortune? Must lame dogs no longer be helped over stiles except through the chilly medium of the rates? And, if so, what about the well of human love which springs as sweet in hearts left solitary by the chances of time, as in the happy mother among her brood? To seek for the wretched and supply their needs, whether individually or by combined action in societies, is Charity, and all the highest impulses of men's nature impel them in this direction. Will there be no outlet in the prosperous future for an altruism which looks beyond the ties of family? Must the lonely rich and the lonely poor be for ever sundered?

The answer is not easy to find, for Utopia lies very far ahead, and even the most primitive and independent community eyed from within, displays all the perplexities which beset the organisers of Charity in densely populated cities. They are no products of modern times. They are inherent in human nature, and doubtless flourished rankly in the infancy of the race.

Yet there are already indications of the place which Charity, never failing, ever greatest of these, will hold among Utopian virtues. In dealing with social problems, she will continue her great experiments. She will serve as taster, if the expression may be permitted, to humanity in altruistic endeavour. In proportion as the State grows more enlightened and Government holds a firmer rein in restraining abuses, the tendency towards conservatism in Public Assistance, which is even now perceptible will become intensified. Methods once approved, even though falling short of conspicuous success, will not be hastily abandoned in favour of theories, however plausible. Level-headed men, elected by popular suffrage, will support the *status quo* and look askance at the enthusiasm of the reformer. If Utopia stands for a State marching towards the perfection of humanity, unobstructed by bad laws and customs, but made up of men moved by the springs of action common to men in the past, it follows that the zeal for humanity will be developed unequally in the Utopians. A small minority, attaining as Utopians a wider outlook, a finer restraint than the modern philanthropist, will still see ahead of their fellows and will count all as loss that is not devoted to their service. It will be their part to perfect the methods in vogue for dealing with the waste products of the nation. They will devote themselves, and make it possible for other men to devote themselves, to the problems affecting men's moral and physical well-being, and under whatever name such experiments may pass in the future, it is certain that Charity is at the root of them all. It is contrary to the principles of sound government to subsidise enterprises which are remote from the interests of the majority, and may even be positively hostile to a minority. But experiments which would be tantamount to social revolutions if undertaken by the State, can be started on a small scale by the philanthropist and tested without risk under varying conditions, even should they yield no result. Such, for instance, are the experiments which have been in progress now for some years under the initiation of private bodies, for the relief of the unemployed. Such is the movement in favour of giving free meals to school children. The arguments against the employment of public moneys for these objects at the outset were too weighty to be overborne. Yet without money it was impossible to ascertain what measure of success might be expected from either movement. Started by voluntary effort, such enterprises, if unsuccessful, fizzle quietly out with the least possible injury to the community. But the State or even the municipality may dislocate trade and rouse the bitterest class antagonism by prematurely undertaking them. It may take generations for private charity to transmute theory into action with such a measure of practical success as shall justify those in authority for throwing the weight of a new benefit on to the shoulders of the people. Thus, if the long series of victories achieved by experimental philanthropy in the history of man is to continue, Charity under the form of the enthusiast for humanity, must be allowed a free hand in Utopia.

And if Charity in its most exalted aspect will be needed in a free Utopia, it will become, under its more simple manifestations, all powerful and ubiquitous. Already there are indications that the old hostile attitude of the rate administrator towards the needy citizen is being abandoned for the tone of brotherhood, which is the essence of Charity. Gradually the relief of the destitute is falling into the hands of the altruistic section of the community—of those who care instead of those who hate. It marks a decisive change all along the line. Methods learned in the great hospitals maintained in the name of Charity are being practised to-day in Poor-law infirmaries which within recent memory were the scene of relentless, if unintentional, cruelty. The altruistic zeal for which there was formerly no outlet but in the cause of Charity is now being pressed into the service of the State, and in workhouses, prisons, asylums, and schools there is a host of enthusiastic workers who look far beyond the immediate emoluments of their post to the amelioration of suffering and the future of the race. It may be that the demands made on the purse by contending philanthropists will cease, and that attempts at social regeneration made by ignorant or self-seeking individuals will be gently thwarted in Utopia. But personal altruistic service will be more highly valued than ever. Utopian administrators will appreciate to the full the spirit of devoted self-sacrifice inspired by the sorrows of others and being themselves imbued with it they will turn this priceless gift to the best possible account for the benefit of their wards.

To conclude.

The new unwalled Utopia lay stretched in all its perfection of detail under the late Twentieth Century sun, and the gracious figure of Charity in flowing robes as pictured in symbolic art, moved pitifully along its streets. A distressful sobbing proceeded from one of the houses, and Charity entered to find a group of little orphans clustered round their dying mother's bed. She stooped to soothe them as the mother expired, and found by her side a practical energetic little lady round whose short skirt the word "Municipality" was inscribed. A notebook was in her hands, and her manner was decided, while yet a sweet light shone in her eyes as she explained, "They will soon be comforted with the foster mother we have chosen for them. I will take them with me, for this is no place for infants." Rebuffed in this quarter, Charity turned with her arms full of loaves to a group of hungry-looking loiterers by the wayside, and again Municipality interposed, "Pray, hold your hand. Relief works are open to these men, and here unless men work they may not eat." Poor Charity moved patiently away to smooth the pillow of a fading consumptive, but even while he gazed at her in gratitude, Municipality entered and carried him off to recover under the shelter of the open sky. Then Charity sought out the blind, and would fain read them words of hope under their affliction, but they had no time to listen, for Municipality had summoned them to school, and they were busy with books and bicycles. Surely the little bed-ridden cripple would respond to tenderness. But at his door was a nurse with an invalid carriage, and he, too, must learn in a building especially planned for his need by Municipality, how to turn his maimed life to account. So Charity found her way at last to the prison, secure of an eager welcome. But within was Municipality busier than ever, growing continually more skilled in the art of strengthening the weak, and turning the energies of the wicked in a new direction. All was activity and regeneration extending far beyond the walls, where a chain of assistance lay ready to guide the backslider to the path of honest citizenship. And Charity went on further, and sought diligently for the infirm of will, the epileptic, the drunken, and the feeble-minded, but she found no trace of them until Municipality came swiftly and led her to one wide-spreading colony after another, where those she deemed unfit to bear children led safe and useful lives under discipline suited to their deficiencies. And Charity learned that many of those considered cured were released from the boundaries of their settlement only to return praying with tears to be shut away once more from the temptations and struggles of the world. Nor was there much prospect that such colonies could ever cease to be needed, since moral failures have been known to subsist in every region and age where history has thrown its light and cannot lack among the crowded populations of modern civilisation. Smiling a little at her own ignorance, and wholly rejoicing Charity made as though she would have passed on her way and left Utopia behind her. But she found again Municipality at her side, grasping her hand with that practical energy which seemed to exclude emotion. And she spoke with a voice of decision.

"Can it be that you fail to recognise me, my Mother? All your experiments, all your labour, all your failures of the past have at length, after these centuries, brought me into being. All that is worthy in me is the spirit you breathed upon me. All that is successful is due to your infinite patience. Stay with me, my Mother, for all that is mine is yours. Leave me not to become corrupted by self-seeking and avarice. Stay, and together we shall prove invincible over sin and want."

So Charity stayed.

